# Editorial: The role of glycolipids and sphingolipids in the differentiation and function of innate immune cells

**DOI:** 10.3389/fimmu.2025.1758633

**Published:** 2025-12-22

**Authors:** Kazuhisa Iwabuchi, Moriya Tsuji, Kohji Kasahara

**Affiliations:** 1 Faculty of Pharmacy, Juntendo University, Urayasu, Japan; 2 Advanced Research Center for Health Drug Discovery, Graduate School of Medicine, Juntendo University, Urayasu, Japan; 3 Institute for Environmental and Gender-specific Medicine, Graduate School of Medicine, Juntendo University, Urayasu, Japan; 4 Aaron Diamond AIDS Research Center, Division of Infectious Diseases, Department of Medicine, Columbia University Vagelos College of Physicians and Surgeons, New York, NY, United States; 5 Biomembrane Group, Tokyo Metropolitan Institute of Medical Science, Tokyo, Japan

**Keywords:** differentiation, glycolipids, immune response, infection, innate immunity, lymphocytes, macrophages, neutrophils

Recent immunology research highlights the essential roles of glycolipids and sphingolipids in directing the differentiation and functions of innate immune cells, including macrophages, neutrophils, and innate T cells. These lipids support pathogen recognition, while many microorganisms exploit their own glycolipids to evade host defenses; for example, Mycobacterium tuberculosis uses mannose-capped lipoarabinomannan to inhibit phagocyte maturation. Innate immune cells have therefore evolved mechanisms to detect and eliminate diverse pathogen-derived glycolipids. Lipid rafts, membrane microdomains enriched in glycolipids and cholesterol, also regulate key signaling processes related to hemostasis and thrombosis. CD1 molecules present lipid antigens to NKT cells, thereby linking innate and adaptive immunity. Despite these advances, deeper insight is needed into how host- and pathogen-derived lipids influence immune cell development and host–pathogen interactions. This Research Topic comprises five original articles and one review article, which collectively describe how glycolipids and sphingolipids regulate innate immune cell differentiation and how pathogen-derived glycolipids affect both innate and cell-mediated immune responses.

Dong et al. reviewed the synthesis and physiological functions of glycosphingolipids (GSLs), as well as their roles in disease and therapeutic applications. Anti-ganglioside and other tumor-associated antibodies show antitumor activity in certain patients. Several viruses, pathogens, and toxins exploit GSLs as cellular receptors. Gangliosides have also been proposed as potential co-receptors or attachment factors that facilitate ACE2-dependent entry of SARS-CoV-2, and inhibitors of glucosylceramide synthase suppress SARS-CoV-2 replication. These findings underscore the utility of GSLs in diagnosing and treating both cancer and infectious diseases.

To determine whether fucosylation defects affect T-cell development intrinsically or via the microenvironment, Yabas et al. characterized mice carrying a mutation in the GDP-fucose regulator GMDS. The Gmds^Y187/Y187 phenotype included significant growth defects and a blockade of T-cell maturation downstream of the double-negative (DN) stage, resulting in a scarcity of DP and SP T cells. Crucially, bone marrow chimeras demonstrated that this failure is hematopoietic-intrinsic; mutant cells could not support T-cell differentiation in competition with wild-type cells, though B-cell lymphopoiesis remained unaffected. The study suggests GMDS functions as an essential cell-autonomous regulator, likely modulating pathways such as Notch.

Neutrophil homeostasis relies on regulated apoptosis, a process modulated by the glycosylated phospholipid phosphatidylglucoside (PtdGlc). PtdGlc is highly expressed on human neutrophils, where it drives differentiation and apoptotic signaling. Previous work by Iwabuchi's group established that targeting PtdGlc with the monoclonal antibody DIM21 triggers early apoptosis. Extending this analysis to the broader neutrophilic lineage (HL-60, KG1, and KG1a), Yokoyama et al. observed that DIM21 sensitivity strictly correlates with PtdGlc presence. The PtdGlc-positive HL-60 and KG1 cells underwent apoptosis upon antibody exposure, while the PtdGlc-negative KG1a line remained resistant. Furthermore, ATRA-induced differentiation in KG1 cells heightened DIM21 susceptibility, resulting in late-stage apoptosis. This pathway bypasses NADPH oxidase and Fas signaling but requires caspase-3 and -8 activation, highlighting a neutrophil-specific, PtdGlc-mediated mechanism with therapeutic potential in AML.

Members of the diverse family of sphingolipids, such as ceramides and sphingosine 1-phosphate (S1P), are bioactive signaling molecules. S1P is carried in blood by its chaperone protein apolipoprotein M (Apo M) on high-density lipoprotein particles. Blaho et al. analyzed sphingolipid levels in plasma and lymph collected from wild-type, ApoM-deficient, and ApoM-transgenic mice to determine how ApoM influences the circulating sphingolipidome. Notably, the study successfully characterized murine lymph fluid, identifying alpha-hydroxyceramide as a major component previously undetected in this compartment.

Severe sepsis frequently progresses to acute respiratory distress syndrome (ARDS), where lung injury is driven by systemic inflammation and the release of neutrophil extracellular traps (NETs). While platelet-mediated NETosis is well established, the specific role of endothelial cells (ECs) has remained unclear, despite their significant dysfunction during sepsis. Fang et al. address this gap by demonstrating that the physical adhesion of neutrophils to ECs is a direct trigger for NET formation. They found that neutrophils firmly adherent to ECs—or ICAM-1–coated surfaces—rapidly generated NETs in response to inflammatory stimuli like LPS or septic plasma. Crucially, blocking the integrin Mac-1 disrupted this process both *in vitro* and *in vivo*, thereby attenuating LPS-induced lung injury.

Study of stroke-associated pneumonia (SAP) is currently limited by animal models that lack the multisystem complexity—spanning neurological, pulmonary, and mucosal immune phenotypes—observed clinically. Here, Wang et al. introduce two rat models of SAP following intracerebral hemorrhage, utilizing either Klebsiella pneumoniae or lipopolysaccharide (LPS) challenge. These models demonstrated severe neurological deficits and tissue damage alongside intestinal dysfunction and a loss of IL-17A+ γδ T cells. In the Klebsiella cohort, 16S rRNA sequencing and metabolomics identified a shift toward opportunistic pathogens and aberrant metabolism, marked by elevated ceramide and quinolinic acid. These findings establish a critical tool for identifying therapeutic targets involving gut microbes.

This Research Topic highlights the impact of glycolipids and sphingolipids, whether originating from host leukocytes or pathogens, on cellular differentiation and immune function. By deciphering these lipid interactions, we gain a clearer picture of innate immunity that can ultimately guide new treatment strategies.

